# Agitation, confusion, and aggression in critically ill traumatic brain injury-a pilot cohort study (ACACIA-PILOT)

**DOI:** 10.1186/s40814-020-00736-5

**Published:** 2020-12-11

**Authors:** David R. Williamson, Sofia Ihsenne Cherifa, Anne Julie Frenette, Mar Saavedra Mitjans, Emmanuel Charbonney, Gabrielle Cataford, Virginie Williams, Julia Lainer Palacios, Lisa Burry, Sangeeta Mehta, Caroline Arbour, Francis Bernard, for the Canadian Critical Care Trials Group

**Affiliations:** 1 Faculté de Pharmacie, Université de Montréal, Montréal, Canada; 2 Research centre, Centre intégré universitaire de santé et de services sociaux du Nord-de-l’île-de-Montréal, Montréal, Canada; 3 Pharmacy Department, Hôpital du Sacré-Cœur de Montréal, Centre intégré universitaire de santé et de services sociaux du Nord-de-l’île-de-Montréal, Montréal, Canada; 4 Faculté de Médecine, Université de Montréal, Montréal, Canada; 5 Critical care, Hôpital du Sacré-Cœur de Montréal, Centre intégré universitaire de santé et de services sociaux du Nord-de-l’île-de-Montréal, Montréal, Canada; 6 Leslie Dan Faculty of Pharmacy, University of Toronto, Toronto, Canada; 7 Pharmacy Department, Mount Sinai Hospital, Toronto, Canada; 8 Department of Medicine, Sinai Health System, and Interdepartmental Division of Critical Care Medicine, University of Toronto, Toronto, Ontario Canada; 9 Faculté de sciences infirmières, Université de Montréal, Montréal, Canada

**Keywords:** Traumatic brain injury, Agitation, Confusion, Aggressiveness, Feasibility, Intensive care

## Abstract

**Background:**

Agitated behaviors are problematic in intensive care unit (ICU) patients recovering from traumatic brain injury (TBI) as they create substantial risks and challenges for healthcare providers. To date, there have been no studies evaluating their epidemiology and impact in the ICU. Prior to planning a multicenter study, assessment of recruitment, feasibility, and pilot study procedures is needed. In this pilot study, we aimed to evaluate the feasibility of conducting a large multicenter prospective cohort study.

**Methods:**

This feasibility study recruited adult patients admitted to the ICU with TBI and an abnormal cerebral CT scan. In all patients, we documented Richmond Agitation Sedation Score (RASS) and agitated behaviors every 8-h nursing shift using a dedicated tool documenting 14 behaviors. Our feasibility objectives were to obtain consent from at least 2 patients per month; completion of screening logs for agitated behaviors by bedside nurses for more than 90% of 8-h shifts; completion of data collection in an average of 6 h or less; and obtain 6-month follow-up for surviving patients. The main clinical outcome was the incidence of agitation and individual agitated behaviors.

**Results:**

In total, 47 eligible patients were approached for inclusion and 30 (64% consent rate) were recruited over a 10-month period (3 patients/month). In total, 794 out of 827 (96%) possible 8-h periods of agitated behavior logs were completed by bedside nurses, with a median of 24 observations (IQR 28.0) per patient. During the ICU stay, 17 of 30 patients developed agitation (56.7%; 95% CI 0.37–0.75) defined as RASS ≥ 2 during at least one observation period and for a median of 4 days (IQR 5.5). At 6 months post-TBI, among the 24 available patients, an unfavorable score (GOS-E < 5 including death) was reported in 12 patients (50%). In the 14 patients who were alive and available at 6 months, the median QOLIBRI score was 74.5 (IQR 18.5).

**Conclusions:**

This study demonstrates the feasibility of conducting a larger cohort study to evaluate the epidemiology and impact of agitated behaviors in critically ill TBI patients. This study also shows that agitated behaviors are frequent and are associated with adverse events.

**Supplementary Information:**

The online version contains supplementary material available at 10.1186/s40814-020-00736-5.

## Key messages regarding feasibility

1. What uncertainties existed regarding the feasibility?

- Recruitment and follow-up rates
- Completion of screening logs for agitated behaviors by bedside nurses
- Time to complete of data collection

2) What are the key feasibility findings?

- Recruitment and follow-up rates were acceptable
- A majority of screening logs were completed by bedside nurses
- The time to complete data collection was satisfactory

3) What are the implications of the feasibility findings for the design of the main study?

- A broadening of inclusion criteria to include patients with a prior history of TBI, neurological disease or major psychiatric illnesses would improve recruitment rates
- Applying a deferred consent model would facilitate recruitment
- Validation of the ABS or any other agitation scale in the ICU population is warranted for future studies

## Introduction

Behaviors such as agitation, confusion, and aggressiveness are problematic in hospitalized intensive care unit (ICU) patients recovering from traumatic brain injury (TBI). These behaviors create substantial risks and challenges for healthcare providers and may delay mobilization and liberation from mechanical ventilation [1, 2]. Although agitated behaviors after TBI have been reported during the early stage of hospital recovery (acute care units and rehabilitation), there are no data specific for the ICU setting [3–6]. In addition, predictors, clinical phenotypes, and impact of agitated behaviors on critically ill TBI outcomes have yet to be described. Hence, there is an urgent need to evaluate the incidence and impact of these behaviors on short- and long-term outcomes in large cohorts. Clinical studies to support evidence-based guidelines for the identification and management of these behaviors are also lacking [7, 8]. Consequently, there is no standard approach to managing these patients, and thus many receive pharmacological (i.e., antipsychotics, sedatives, or analgesics) and non-pharmacological (i.e., restraints) interventions that may not be helpful or may adversely impact short and long-term recovery [7, 9–12]. There is a general consensus regarding the urgent need for clinical studies evaluating optimal strategies for the management of agitated behaviors in TBI patients admitted to ICU [8]. Prior to a multicenter observational cohort study, the feasibility of recruitment and adherence to study procedures needs to be assessed. Pilot studies are essential to assess the feasibility of conducting a larger study and increase the probability of success of the main study [13]. In this pilot study, we aimed to evaluate the feasibility of conducting a large multicenter prospective observational cohort evaluating the epidemiology and impact of agitated behaviors in critically ill TBI patients.

## Methods

### Study design and consent

This was a prospective, single-center pilot cohort study of adult patients admitted to the ICU with a TBI. Consent was obtained from the patient or their surrogate. If consent was initially obtained from a surrogate, patient consent was obtained once he or she was judged competent. The protocol was approved by the local research ethics board.

### Feasibility aims and clinical outcomes

The primary objective was feasibility as assessed with the following goals: (1) to obtain consent from at least 2 patients per month; (2) completion of screening logs for agitated behaviors by bedside nurses for more than 90% of 8-h shifts; (3) completion of data collection in an average of 6 h or less; (4) obtain 6-month follow-up for surviving patients. The secondary outcomes were (1) the incidence of agitation, defined as a Richmond Agitation and Sedation Scale (RASS) [14] score of 2 or more at least once during the ICU stay, as well as the incidence of individual agitated behaviors, measured with an observation log designed for the study (Additional file 1); (2) self-harm (i.e., self-extubation, catheter removal); (3) ICU-free days and hospital length stay; (4) hospital mortality, and (5) 6-month functional outcome and quality of life. We did not set pre-specified criteria for success to 6-month follow-up.

### Study setting

The study was conducted in the 36-bed ICU of Sacré-Coeur Hospital, a University-affiliated teaching hospital and level 1 trauma center in Montreal, Canada.

### Eligibility criteria

Patients 18 years and older admitted to the ICU with TBI (severe, moderate, or mild) and an abnormal cerebral CT scan, screened within 48 h of ICU admission to enable the description of a maximum of agitated behaviors, and had an expected stay of more than 48 h (as confirmed with attending ICU physician) were eligible for inclusion. We excluded patients in whom agitated behaviors could be difficult to evaluate because of muscle function loss (i.e., paraplegia or quadriplegia) or was potentially already an issue prior to admission [prior history of TBI or major neurological disease with sequelae (i.e., Parkinson’s, neuroinfections), stroke history of major psychiatric disease (i.e., schizophrenia, major depression, bipolar disorders, schizoaffective disorders), and prior history of cognitive dysfunction]. We also excluded patients at high risk of short-term mortality (Child C liver cirrhosis, chronic heart failure NYHA class IV, end-stage renal or chronic respiratory disease, malignancy with life expectation less than 1 year, and anticipated withdrawal of advanced life support).

### Patient recruitment

Between September 2018 and July 2019, we screened all new ICU admissions for study eligibility from Monday to Friday. Eligible patients or their substitute decision maker were approached for informed consent.

### Procedures

Baseline data included age, sex, level of education, co-morbidities (psychiatric disease, chronic pain), medication history prior to hospital admission, smoking status, current drug-abuse, or alcohol abuse (self-reported or family reported). We defined chronic alcohol use as the consumption of more than 2 drinks per day or equivalent of 750 ml 40% alcohol per week; and the use of recreational drugs including marijuana as at least once in the week prior to admission [15]. We collected data on the type of trauma (motor-vehicle accident, falls, violence, sports-related), concomitant injuries (limb fractures, thorax, abdomen), admission Acute Physiology and Chronic Health Evaluation (APACHE II) score, and Injury Severity Score (ISS) [16, 17]. The severity of TBI was documented using the ICU admission unsedated Glasgow Coma Scale (GCS) score and classed into one of three severity groups (mild with a GCS of 13 to 15, moderate with a GCS of 9 to 12 and severe with a GCS of 8 or less) [18]. A neurointensivist (FB) reviewed all head CT scans using the Marshall and Rotterdam scores [19, 20]. During ICU stay, we collected daily clinical parameters (RASS [14], pain scores, GCS, intracranial pressure, mean arterial pressure, and cerebral oxygenation), medications (i.e. sedatives, analgesics, vasopressors, anticonvulsants, antipsychotics), environmental variables (room type, visitors, window presence), physical restraint use, and mobilization.

In all patients, we documented 14 agitated behaviors every 8-h nursing shift using a dedicated observation tool. We documented 11 behaviors from the Agitated Behavior Scale (inattention, impulsiveness, uncooperative, violent behavior, explosive or unpredictable anger, self-stimulating behavior, pulling at tubes or restraints, restlessness, repetitive behavior, emotional instability, and inappropriate speech) [21]. Given the ICU context and disease acuity, three behaviors from the Agitated Behavior Scale were not documented, as we judged they would be rare or difficult to evaluate in neurologically impaired critically ill patients (wandering, sudden changes of mood and self-abusiveness). These behaviors were replaced with disorientation, hallucinations/delusions, and fighting the ventilator based on the Intensive Care Delirium Screening Checklist and RASS score [14, 22]. Bedside nurses documented the severity of behaviors, the interventions used to control the behaviors (re-orientation, constant supervision, physical restraint, environmental modifications, and pharmacological interventions), and frequency of treatment interference including accidental removal of catheters and other medical devices. Severity of behaviors was defined as mild if the behavior was present but did not prevent the conduct of appropriate behavior, moderate if the patient needed to be redirected from agitated to an appropriate behavior, and extreme when the behavior interfered with patient care and continued despite interventions including reorientation [21]. Given the suboptimal performance of delirium screening tools in previous studies of TBI patients, we did not measure delirium [23, 24].

Prior to starting the study, training sessions were held for all three nursing shifts. These sessions included a review of the study protocol and training for agitated behavior documentation logs. On a daily basis, research staff ensured comprehension of agitated behavior documentation logs by bedside nurses for each enrolled patient. To ensure accurate capture and comprehension of agitated behaviors, when feasible we filmed patients for up to four 1-h periods on separate days following the weaning of sedatives. Two investigators independently reviewed the videos and recorded the presence or absence of the 7 behaviors which could be easily evaluated with the videos, as well as the severity. We compared the patient behavior assessment of the 2 investigators, and between the investigators and bedside nurses documentation logs in order to assess optimal completion of the observation tool [25]. For the videos, we obtained consent from bedside nurses and any other healthcare provider who was likely to be filmed (i.e., respiratory therapists, orderlies). In the event of a patient/substitute decision-maker withdrawing consent, all videos were electronically destroyed. Screening time and time to complete the study forms were documented for the first 10 enrolled patients.

Patients were followed until one of the following events: ICU discharge, 28 days, or death. Six months following study inclusion, we contacted surviving patients (or their families in the instance where patients were unable to self-report) by telephone for functional and health-related quality of life outcomes. We evaluated functional outcome with the Glasgow Outcome Scale Extended (GOS-E) and health-related quality of life with the Quality of Life after Brain Injury (QOLIBRI) instrument [26, 27]. The GOS-E is a functional outcome scale that has 8 levels of patient status ranging from a minimum of 1 (dead) to 8 (upper good recovery). The QOLIBRI is a health-related quality of life instrument specific for TBI and is reported on a scale of 0 to 100, with 0 being the worst possible quality of life and 100 the best.

### Statistical analysis

A total of 30 patients were expected to be recruited in this pilot study [28]. Simple descriptive statistics were used to report feasibility outcomes. In order to establish reliability of the agitated behavior documentation logs, raw agreement (the proportion of overall agreement) among two investigators and bedside nurses was measured for 7 behaviors recognizable on video (agitation, pulling on tubes and catheters, self-stimulating behavior, repetitive behavior, uncooperative, violent behavior, anger). In addition, agreement with the degree of the behavior among the investigators and bedside nurses was measured using weighted kappa. To summarize the level of agitated behaviors, we summed the total scores of the behaviors (mild = 1, moderate = 2, and extreme = 3).

Continuous variables were described using measures of central tendency and spread (means and SD, or median and interquartile range depending on data distribution). Frequencies, proportions, and 95% CIs were used to describe categorical variables. Student’s *t* tests or Mann–Whitney *U* test, if data was skewed, were used to compare continuous variables, and chi-square or Fisher’s exact tests to compare categorical variables.

## Results

### Recruitment and feasibility

**Fig. 1:**
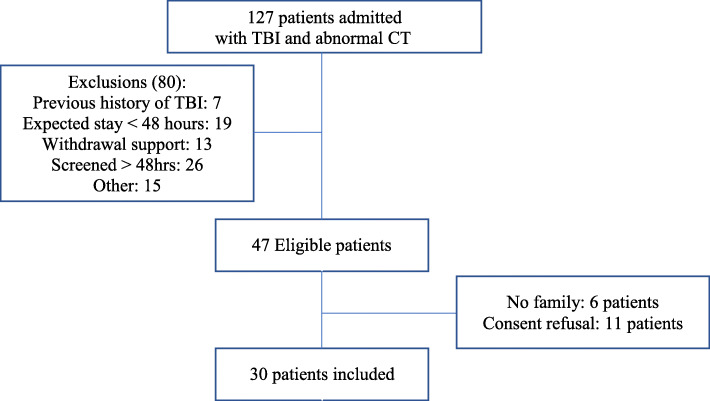
Patient flow chart

**Fig. 2:**
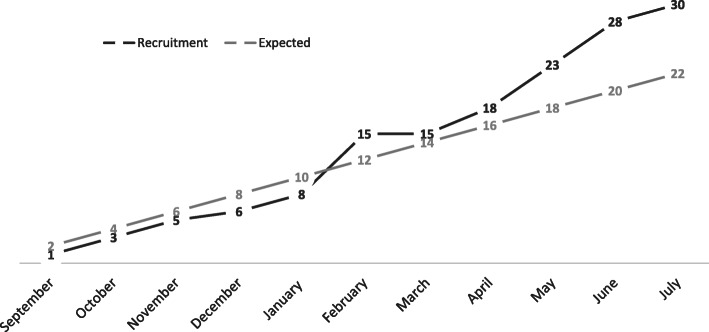
Recruitment rates

During the 10-month study period, 127 TBI patients were screened and 47 (37%) were eligible (Fig. 1). The main reasons for exclusion were being screened more than 48 h following ICU admission (26 patients), an expected stay of less than 48 h (19 patients) and anticipated withdrawal of treatments (13 patients). Of the 47 patients approached for inclusion, 11 patients (23.4%) declined participation and in 6 patients (12.8%), no substitute decision-maker was available for consent, leaving a cohort of 30 patients (63.8% consent rate). During the study, no patient or substitute decision-maker withdrew consent. The study recruitment rate was 3 patients per month, in line with the 2 patient per month goal (Fig. 2). Daily screening required an average of 40 min per day and research assistant data collection took an average of 4 h per patient.

In total, 96% (794 out of 827 possible 8-h periods) of agitated behavior logs were completed by bedside nurses, with a median of 24 observations (IQR 28.0) per patient. For one patient, no behavior logs were completed as the patient was discharged within 8 h of recruitment. A total of 38 videos in 19 patients (varying from 1 to 4 videos per patient) were filmed and independently reviewed by 2 investigators. The raw agreements between the two investigators and bedside nurses for the seven behaviors examined were 81% and 76%, respectively. The weighted kappa for the behavior severity was fair with 0.271 (95% CI 0.149–0.393) and 0.255 (95% CI 0.127–0.382) for evaluators 1 and 2, respectively. The weighted kappa was moderate between the two investigators with 0.44 (95% CI 0.32–0.56). At 6 months, GOS-E and QOLIBRI were obtained for 24 (80.0%) and 14 patients (46.7%), respectively. Four patients were not reachable after multiple attempts and two refused participation, while nine patients had died and one was in a vegetative state.

### Baseline characteristics of the cohort

As presented in Table 1, the median age was 64.5 years (IQR 41.3), 73.3% were men, and the most frequent cause of TBI was falls (50%) followed by motor vehicle accidents (43%). TBI was mild, moderate, and severe in 27%, 43%, and 30% of cases respectively. Two (6.7%) and 5 (16.7%) patients abused alcohol or actively used recreational drugs respectively. Four patients (12.9%) were actively medicated for attention deficit hyperactivity disorder (ADHD), 9 patients (29.0%) were treated for hypertension, and 5 (16.1%) were diabetic.

**Table 1:** Demographics

Demographics	Agitation ***N*** = 17	No agitation ***N*** = 13	All patients ***N*** = 30
Median age, years (IQR)	60 (46.5)	66.0 (39)	64.5 (41.3)
Median APACHE 2 (IQR)	16.0 (9.0)	17.0 (10.5)	16.5 (9.25)
Median Injury Severity Score (IQR)	26.0 (14.0)	22.0 (26.5)	26.0 (18.0)
ICU admission GCS	6.0 (IQR 4.5)	13.0 (IQR 11.5)	6.0 (10.3)
Male sex	15 (88.2%)*	7 (53.8%)	22 (73.3%)
Primary language at home			
French	13 (76.4%)	12 (92.3%)	25 (83.3%)
English	1 (5.9%)	1 (7.7%)	2 (6.7%)
Other	3 (17.6%)	0	3 (10.0%)
Highest education level completed			
Primary	5 (29.4%)	3 (23.1%)	8 (25.8%)
Secondary	10 (58.8%)	7 (53.8%)	16 (51.6%)
University	2 (11.8%)	3 (23.1%)	5 (16.1%)
TBI severity			
Severe	4 (23.5%)	5 (38.5%)	9 (30.0%)
Moderate	11 (64.7%)	2 (15.4%)	13 (43.3%)
Mild complex	2 (11.8%)	6 (46.2%)	8 (26.7%)
TBI mechanism			
Falls	9 (52.9%)	6 (46.2%)	15 (50%)
MVA	7 (41.2%)	6 (46.2%)	13 (43%)
Other	1 (7.7%)	1 (5.9%)	2 (7%)
Localization of lesions			
No lesions	1 (5.9%)	2 (15.4%)	3 (10%)
Frontal	8 (47.1%)	3 (23.1%)	11 (36.7%)
Temporal	0 (0%)	1 (7.7%)	1 (3.3%)
Fronto-temporal	5 (29.4%)	7 (53.8%)	12 (40%)
Parieto-occipital	10 (58.8%)	4 (30.8%)	14 (46.7%)
Marshall score, median (range)	2 (1-6)	1 (1-5)	1.5 (1-6)
Rotterdam score, median (range)	3 (1-5)	3 (2-6)	3 (1-6)
Hearing impairment	3 (17.6%)	2 (15.4%)	5 (16.7%)
Attention deficit hyperactivity disorder	4 (23.5%)	0	4 (13.3%)
Ethanol positive screening on admission	4 (23.5%)	3 (23.1%)	7 (23.3%)
Active alcohol use	2 (11.8%)	0	2 (6.7%)
Active drug use (< 7 days)*	5 (29.4%)	0	5 (16.7%)
Active smoking	6 (35.3%)	4 (30.8%)	10 (33.3%)

Data are presented as median (IQR), or *N* (%) unless otherwise stated. *P < 0.05; other comparisons are non-significant

*APACHE* Acute Physiology and Chronic Health Evaluation, *ICU* intensive care unit, *IQR* interquartile range, *GCS* Glasgow Coma Scale, *MVA* motor vehicle accident, *TBI* traumatic brain injury

### Agitation

During the ICU stay, 17 of 30 patients developed agitation (56.7%; 95% CI 0.37–0.75%) defined as a RASS ≥ 2 during at least one observation period. In these 17 patients, RASS ≥ 2 was reported for a median of 4 days (IQR 5.5), while RASS < 2 was reported for a median of 5 days (IQR 8.5). In comparison to patients who did not develop agitation, patients with agitation were more often male, had moderate TBI, lower median GCS scores, were active drug or alcohol abusers, and were receiving treatment for a diagnosis of ADHD (Table 1). Patients with agitation were more likely to receive mechanical ventilation (14/17 patients; 82.4%) versus (6/13 patients; 46.2%) but this difference did not reach statistical significance (*p* = 0.056).

### Agitated behaviors

**Fig. 3:**
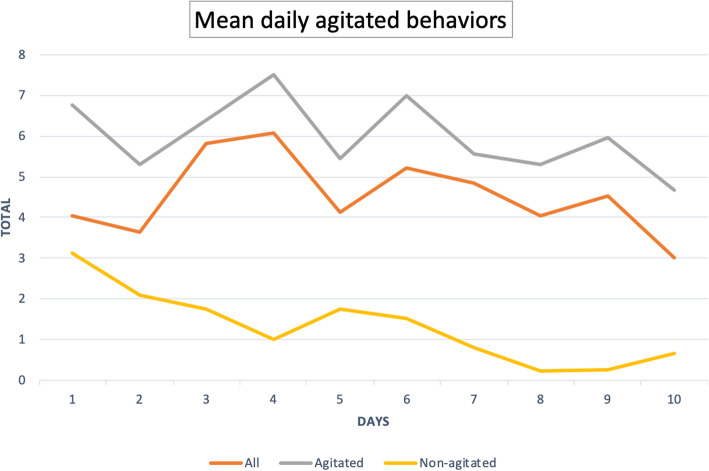
Mean daily agitated behaviors

The proportion of the individual agitated behaviors per observation period is described in Table 2. The most common behavior was restlessness, present in 385 observation periods (48.5%) and 19 patients (65.5%) during the ICU stay. Restlessness was reported by bedside nurses as moderate to extreme in 219 observation periods (27.6%) and seemed more common during night shifts (53.5%) compared to daytime (46.5%) and evening shifts (45.7%) (*p* = 0.14). Other common behaviors manifested by more than 50% of patients at least once during the ICU stay included inattention (65.5%), pulling on tubes and catheters (62.1%), disorientation (58.6%), self-stimulating behavior (55.2%), and uncooperativeness (51.7%). Violent behavior (31.0% of patients) and anger (20.7% of patients) were also commonly reported and more frequent during night-time shifts. When examining the co-occurrence of behaviors, in the 19 patients presenting with restlessness, bedside nurses reported pulling on tubes and catheters (69.7%), uncooperativeness (45.3%), and impulsiveness (43.0%) during the 8-h observation period. The mean daily sum of the 14 behaviors in all patients was 4.6 (SD 6.6) over the first 10 days of ICU stay and was greatest on days 4 and 6 of ICU stay (Fig. 3).

**Table 2:** Individual agitated behaviors per shift, severity and patient

Behaviors	Day (%) ***N*** = 271	Evening (%) ***N*** = 267	Night (%) ***N*** = 256	Total (%) ***N*** = 794	Moderate–extreme (%) ***N*** = 794	% Patients ***N*** = 29
Restlessness	46.5	45.7	53.5	48.5	27.6	65.5
Inattention	18.8	24.3	24.6	22.5	14.6	65.5
Pulling on tubes and catheters	34.7	40.8	39.1	38.2	21.7	62.1
Disorientation	28.4	27.7	28.9	28.3	19.2	58.6
Self-stimulating behavior	13.6	18.4	21.1	17.6	7.9	55.2
Uncooperative	17.3	24.0	30.9	23.9	12.0	51.7
Repetitive behavior	20.3	19.1	24.2	21.2	9.6	48.3
Impulsiveness	21.8	22.8	23.0	22.5	10.3	44.8
Inappropriate speech	10.0	12.4	19.1	13.7	6.8	37.9
Violent behavior	11.8	10.1	17.2	13.0	4.3	31.0
Fights ventilator	5.2	7.9	6.3	6.4	2.6	24.1
Anger	8.9	10.1	15.6	11.5	3.1	20.7
Emotional instability	9.2	9.4	13.7	10.7	4.0	20.1
Hallucinations/delusions	5.2	7.8	10.2	7.7	0.9	20.1

Data presented as percentage of patients

### Interventions

Physical restraints were used on 18 patients (60%), mostly in patients who developed agitation (16 of 17 patients who developed agitation during ICU stay; 94.1%). For the management of restlessness, nurses reported interventions in 303 of 385 (78.7%) observation periods. A pharmacological intervention was used in 212 patients (55.1%). Antipsychotics, opiates, dexmedetomidine, propofol, and benzodiazepines were used in 20.8%, 19.0%, 13.2%, 10.8%, and 8.1% of cases, respectively. Physical restraints and environmental modifications such as diming the lights or reducing noise level were used on 66 (21.8%) and 25 (8.3%) occasions.

### Clinical outcomes

Overall, 20 patients (66.7%) were mechanically ventilated during the ICU stay, for a median duration of 5 days (IQR 6.5). The median ventilator-free days within 28 days after ICU-admission was 24.5 days (IQR 8.5) in the entire study cohort, 23 (IQR 4.5) in the agitated group and 28 (IQR 28) in the non-agitated group. Accidental removal of nasogastric tubes, peripheral venous catheter removal and wound dressings were the most common types of interference associated with agitation, occurring in 4 (13.3%), 3 (10.0%), and 2 (6.7%) patients, respectively. Among other significant clinical events, accidental extubation and fall were described in one patient each, both of whom were agitated. The median length of ICU stay was 8.5 days (IQR 11.5) and the median ICU-free days within 28 days (i.e., days not in the ICU within 28 days of admission) of ICU admission was 18 days (IQR 18.8). ICU, hospital, and 6-month mortality were 13.3% (4/30), 23.3% (7/30), and 30% (9/30), respectively. Hospital mortality was greatest in patients with moderate (2 of 13 patients) and severe TBI (5 of 9 patients).

In total, 2 (11.8%) and 5 (38.5%) patients in the agitated and non-agitated groups died, respectively. Five (29.4%) and 4 (30.8%) patients in the agitated and non-agitated groups were discharged directly home whereas 5 (29.4%) and 3 (23.1%) were transferred to a rehabilitation hospital, respectively. Five patients (29.4%) in the agitated group and one patient (7.7%) in the non-agitated groups were transferred back to their referring hospital. In the 17 patients who developed agitation, the median ICU-free days within 28 days of ICU admission was 17 days (IQR 12.0) compared to 22 days (IQR 25.5) in patients without agitation.

At 6 months post-TBI, among the 24 available patients, an unfavorable score (GOS-E < 5 including death) was reported in 12 patients (50%). In the 12 remaining patients, low moderate disability, upper moderate disability, low good recovery, and upper good recovery were reported in 2 (8.3%), 3 (12.5%), 4 (16.7%), and 3 (12.5%) patients, respectively. Of the 15 patients with agitation having GOS-E results, 7 (46.7%) had an unfavorable outcome compared to 5 of 9 patients (55.6%) without agitation. In the 14 patients who were alive and available at 6 months, the median QOLIBRI score was 74.5 (IQR 18.5).

## Discussion

In this pilot study, we demonstrated the feasibility of conducting a prospective observational study on agitated behaviors in critically ill TBI patients. In our pilot study, we obtained an adequate three-patient per month recruitment rate as well as screening and data collection times of a mean less than 6 h. In addition, the measure of agitated behaviors by ICU nurses was satisfactory with a high proportion of behavioral observation logs being completed, and an acceptable agreement between investigators and bedside nurses for observed behaviors was attained. The fair agreement may be partially explained by the limits of punctual 1-h videos as bedside nurses may have the better knowledge of patient behaviors that allow to better classify them and evaluate their severity. It may also be that bedside nurses, having a better knowledge of their patients, were in a better position to classify behaviors. Research assistant may also have been more stringent in applying behavior criteria. The usefulness of short videos for future studies may be limited. A larger study would need to provide in-depth training of bedside nurses with frequent reminders and the use of teaching tools such as video descriptions of the behaviors. In general, we found nurses to be enthusiastic about the project, most often because they perceived importance of the research question being studied.

We also identified numerous strategies for recruitment and methods modifications to improve our protocol for a definitive study. Despite a satisfactory recruitment rate, we identified opportunities to improve recruitment rates including a broadening of inclusion criteria to recruit patients with a prior history of TBI, neurological disease, or major psychiatric illnesses. The broadening of inclusion criteria would offer better description of the true incidence of agitated behaviors. In addition, as availability of family members for consent within 48 h of admission was a challenge, applying a deferred consent model would facilitate recruitment. A priori informed consent was mandated by our research ethic board because of the use of videos. A future multicenter study would not include videos, and given that video was a common reason for consent declination this should facilitate patient recruitment. A waived-consent model should also be considered to improve the external validity of findings. As for the study methods, nurses did comment that the number of behaviors collected was a time burden and efforts should be made to reduce them. Using a shorter version of the QOLIBRI could also simplify procedures.

Clinically, we observed a high incidence of agitation defined as RASS ≥ 2 (56.7%). Individual behaviors such as restlessness, inattention, pulling on tubes and catheters, disorientation, self-stimulating behavior, and uncooperativeness were observed in more than 50% patients. The incidence of agitation was similar to previous studies in non-TBI ICU patients that reported an incidence of 31.8 to 59% using the RASS and Ramsay score [1, 29, 30]. However, agitation seemed greater than in previous studies of TBI patients in other settings which reported an incidence of 19 to 41% [6, 31–33]. Anger (20.7%) and violent behavior (31.0%), which can cause distress and be dangerous for bedside health care workers, were also reported in an important proportion of patients, most often during night shifts. For this feasibility trial, we did not plan to document psychological or physical consequences for bedside healthcare workers. There may be an opportunity to collect this data in a larger study. As suspected, agitation was associated with self-harm (e.g., nasogastric tube and peripheral catheter removal, falls, and accidental extubation) and decreased ICU-free days within 28 days. Individual behaviors such as restlessness were most often managed with pharmacological agents (antipsychotics, analgesics, and sedatives) and physical restraints.

Although not the objective of this study, we did observe factors associated with agitation. As in previous studies of non-TBI patients, a history of drug or alcohol use was associated with agitation [1, 30]. In addition, moderate TBI patients were at greater risk of developing agitation, in part because many severe TBI patients never regained enough consciousness to develop agitation. Interestingly, all patients receiving treatment for ADHD developed agitation during the ICU stay. Male sex was also identified as a risk factor, a finding which may be confounded by factors such as substance abuse and ADHD, which were more common in men. An adequately powered cohort study would enable the evaluation of potential predictors in multivariate models and identify modifiable risk factors.

The strengths of this study include prospective behavior documentation by bedside nurses, who are better suited to observe these events than research personnel; and the use of videos to assess documentation of behaviors with the observation tool. The study also estimated the incidence of agitation and individual behaviors in ICU patients, informing future clinical studies. This study also has limitations, including being conducted in a single center. Hence, feasibility in other research sites may be different. As identified with the videos, the intensity scoring for the behaviors may have been suboptimal and additional training will be required for future studies to ensure optimal comprehension of definitions. Although well validated for the evaluation of sedation and agitation in ICU patients, the RASS has not been extensively studied in neurocritical care patients [34]. We opted not to use the ABS to define agitation as it had not been validated in the ICU setting. We also only reported a proportion of the behaviors from the ABS, limiting the psychometric properties of the scale and its capability of measuring agitation. Validation of the ABS or any other agitation scale in the ICU population is warranted for future studies. Finally, this pilot study was not powered to evaluate risk factors and clinical outcomes and thus should be regarded as hypothesis generating.

## Conclusion

In this pilot study, we demonstrated the feasibility of conducting a larger cohort study to evaluate the epidemiology and impact of agitated behaviors in critically ill TBI patients, as well as identified opportunities for protocol improvement. We found that agitated behaviors are frequent in the ICU following TBI and are associated with adverse events, including accidental device removal. Potential risk factors include male sex, substance abuse, ADHD, and moderate TBI.

## Supplementary Information

**Additional file 1.**

## Data Availability

The datasets during and/or analyzed during the current study are available from the corresponding author on reasonable request.

## Abbreviations

ICU: Intensive Care Medicine
ISS: Injury Severity Score
RASS: Richmond Agitation Sedation Scale
TBI: Traumatic brain injury
