# Supplementary material for: Agitation, confusion, and aggression in critically ill traumatic brain injury-a pilot cohort study (ACACIA-PILOT)
Source: Pilot Feasibility Stud. 2020 Dec 11;6:193. doi: 10.1186/s40814-020-00736-5 (PMC7729148; doi:10.1186/s40814-020-00736-5)
Supplement: Supplementary file 1 — Additional file 1. [file 40814_2020_736_MOESM1_ESM.docx]

Appendix 1

**Table 1: Agitated behaviour data collection form**

Please document the following behaviours once every 8 hour-shift

| Behaviours | Definitions | Intensity* | Interventions needed |
| --- | --- | --- | --- |
| **Agitation** | | | |
| **Restlessness** | Restlessness, pacing, excessive movement. |  |  |
| **Impulsiveness** | Impulsive, impatient, low tolerance for pain or frustration. |  |  |
| **Self-stimulating behaviour** | Rocking, rubbing, moaning or other self-stimulating behaviour. |  |  |
| **Repetitive behaviour** | Repetitive behaviours, motor and/or verbal. |  |  |
| **Pulling** | Pulling at tubes, restraints, etc. |  |  |
| **Uncooperative** | Uncooperative, resistant to care, demanding. |  |  |
| **Fights ventilator** | Patient–ventilator dyssynchrony |  |  |
| **Confusion** | | | |
| **Inattention** | Short attention span, easy distractibility, and inability to concentrate. |  |  |
| **Disorientation** | Any obvious mistake in time, place or person |  |  |
| **Emotional instability** | Sudden changes of mood, easily initiated or excessive crying and/or laughter. |  |  |
| **Hallucination/ delusions** | The unequivocal clinical manifestation of hallucination or of behaviour probably due to hallucination (e.g., trying to catch a non-existent object) or delusion. |  |  |
| **Inappropriate speech** | Inappropriate, disorganized or incoherent speech. Inappropriate display of emotion related to events or situation. Rapid, loud or excessive talking. |  |  |
| **Aggressiveness** | | | |
| **Violent behaviour** | Violent and/or threatening violence toward people or property. (verbal or physical) |  |  |
| **Anger** | Explosive and/or unpredictable anger. |  |  |

**Intensity levels:**

1-Mild degree: behaviour does not prevent the conduct of other appropriate behaviors.

2-Moderate degree: the individual needs to be redirected from an agitated to an appropriate behavior and the response is positive.

3-Extreme degree: agitated behaviour interferes with therapies and remains even when external cueing or redirection is provided.

**Interventions:**

Document the interventions needed to manage the agitated behaviour including: redirection and reorientation, bedrails, physical restraints (specify type: wrist, ankle, waist, mitts or other), constant observer at bedside, pharmacological intervention (specify agent: antipsychotics, sedatives, opiates, others), environmental modifications (lighting, noise, etc.) or any other intervention.
